# Supplementary material for: DMRforPairs: identifying Differentially Methylated Regions between unique samples using array based methylation profiles
Source: BMC Bioinformatics. 2014 May 15;15:141. doi: 10.1186/1471-2105-15-141 (PMC4046028; doi:10.1186/1471-2105-15-141)
Supplement: Additional file 2 — DMRforPairs output for the comparison of A431-MCF7 and NA17018-NA17105. Please start from the HTML files in each folder. Available via the BMC Bioinformatics website. [file 1471-2105-15-141-S2.zip › 1394847754114233_MOESM2_ESM/A431_MCF7/figures/34890.pdf]

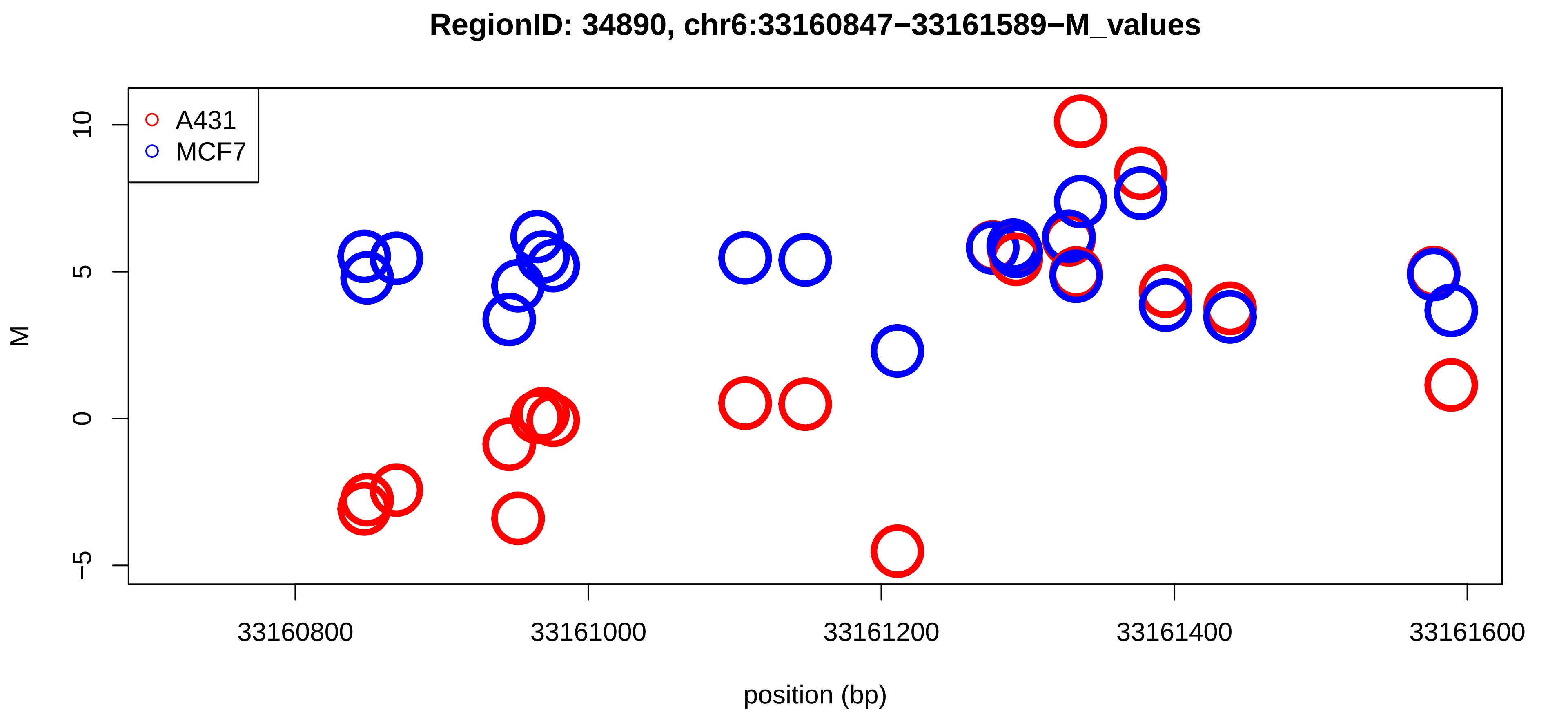
RegionID: 34890, chr6:33160847−33161589−M_values
A431
MCF7
M
10
5
0
−5
33160800
33161000
33161200
33161400
33161600
position (bp)

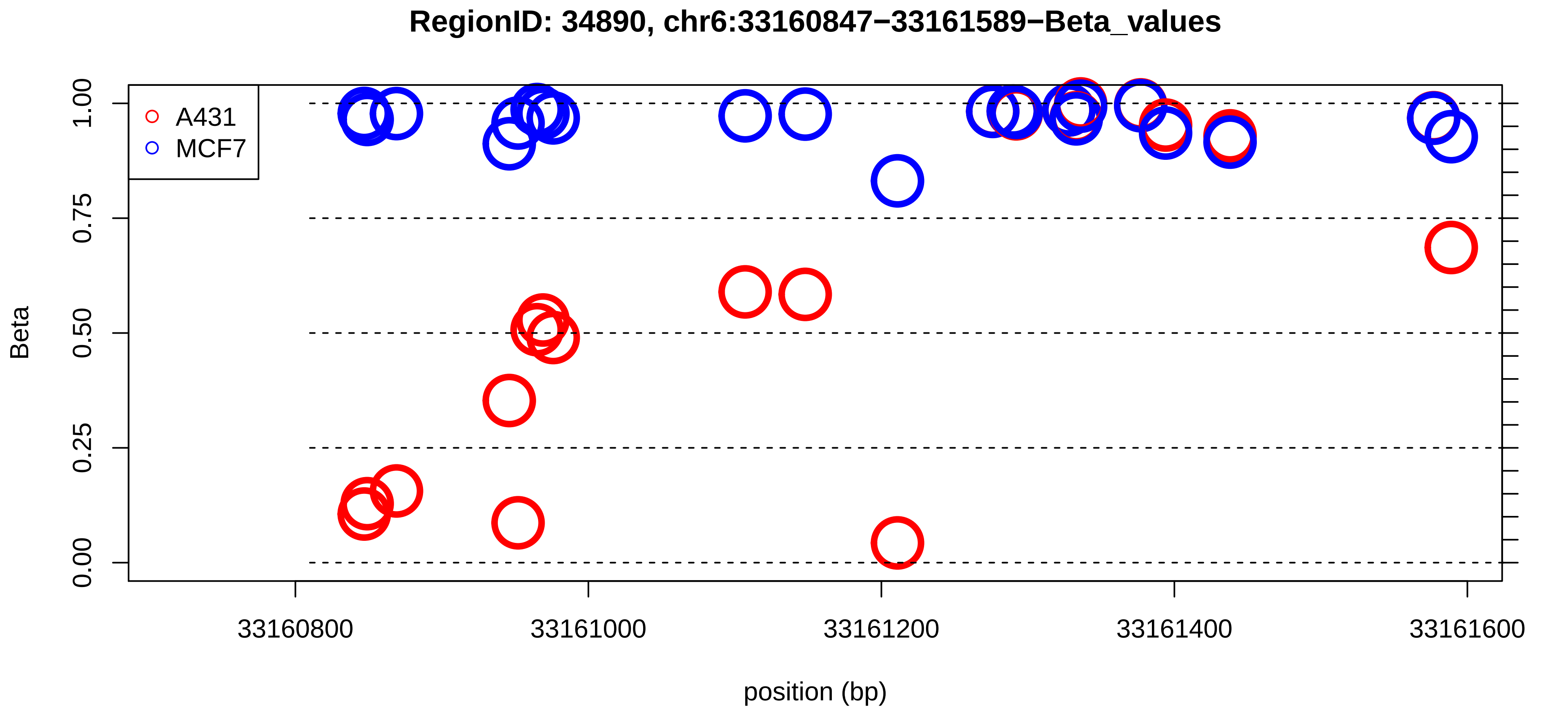
RegionID: 34890, chr6:33160847−33161589−Beta_values
A431
MCF7
Beta
1.00
0.75
0.50
0.25
0.00
33160800
33161000
33161200
33161400
33161600
position (bp)

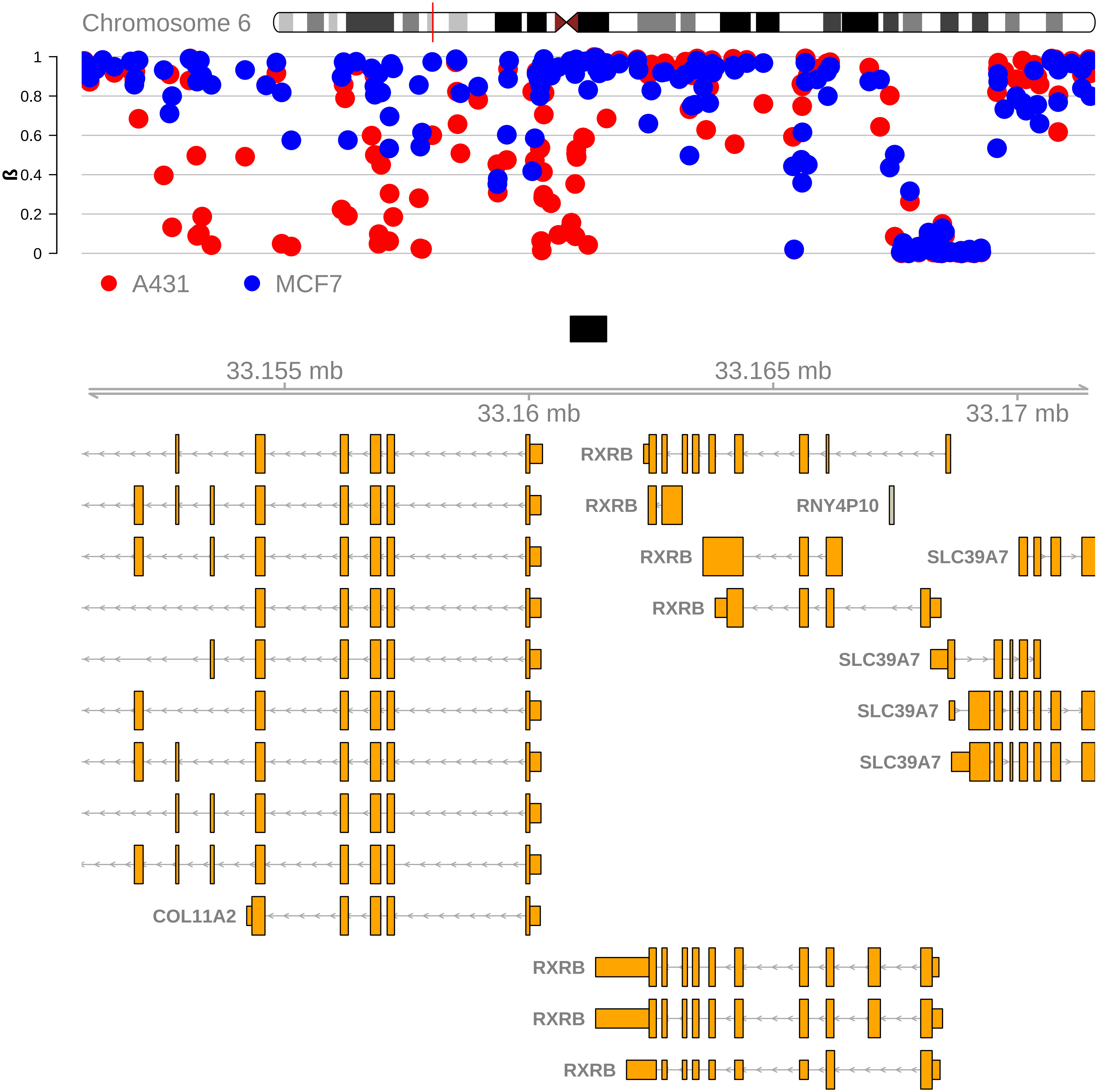

Chromosome 6
1
0.8
0.6
ß 0.4
0.2
0
A431
MCF7
33.155 mb
33.165 mb
33.16 mb
33.17 mb
RXRB
RXRB
RNY4P10
RXRB
SLC39A7
RXRB
SLC39A7
SLC39A7
SLC39A7
COL11A2
RXRB
RXRB
RXRB
